# A multicenter single‐arm trial of neoadjuvant pyrotinib and trastuzumab plus chemotherapy for HER2‐positive breast cancer

**DOI:** 10.1002/mco2.435

**Published:** 2023-12-07

**Authors:** Qiyun Shi, Xiaowei Qi, Peng Tang, Linjun Fan, Li Chen, Shushu Wang, Yan Liang, Ying Hu, Minghao Wang, Lin Ren, Guozhi Zhang, Xuanni Tan, Long Yuan, Junze Du, Xiujuan Wu, Mengyuan Wang, Hongying Che, Pengwei Lv, Dejie Chen, Jinhui Hu, Qiuyun Li, Yanwu Zhang, Kunxian Yang, Yuan Zhong, Chuang Chen, Zemin Zhou, Liyuan Qian, Jingwei Zhang, Mingde Ma, Yi Sun, Yi Zhang, Jun Jiang

**Affiliations:** ^1^ Department of Breast and Thyroid Surgery Southwest Hospital, Army Medical University Chongqing China; ^2^ The Eighth Medical Center of Chinese PLA General Hospital Beijing China; ^3^ Department of Breast Surgery Chongqing University Three Gorges Hospital Chongqing China; ^4^ Department of Thyroid and Breast Surgery Zigong First People's Hospital Zigong Sichuan China; ^5^ Department of Breast surgery The First Affiliated Hospital of Zhengzhou University Zhengzhou Henan China; ^6^ Department of General Surgery Xiangyang Central Hospital Xiangyang Hubei China; ^7^ Department of Breast Surgery The First Hospital of Hunan University of Chinese Medicine Changsha Hunan China; ^8^ Department of Breast Surgery Guangxi Medical University Cancer Hospital Nanning Guangxi China; ^9^ Department of Breast Surgery The Third Affiliated Hospital of Zhengzhou University Zhengzhou Henan China; ^10^ Department of Breast and Thyroid Surgery The First People's Hospital of Yunnan Province Kunming Yunnan China; ^11^ Department of Breast and Thyroid Surgery The Central Hospital of Wuhan Wuhan Hubei China; ^12^ Department of Breast and Thyroid Surgery Hubei General Hospital Wuhan Hubei China; ^13^ Department of Breast and Thyroid Surgery Huaihua First People's Hospital Huaihua Hunan China; ^14^ Department of Breast and Thyroid Surgery The Third Xiangya Hospital of Central South University Changsha Hunan China; ^15^ Department of Breast and Thyroid Surgery Zhongnan Hospital of Wuhan University Wuhan Hubei China; ^16^ Department of Thyroid and Breast Surgery Huaihe Hospital of Henan University Kaifeng Henan China; ^17^ Department of Breast and Thyroid Surgery Xuchang Central Hospital Xuchang Henan China

**Keywords:** HER2‐positive breast cancer, multicenter study, neoadjuvant therapy, pyrotinib, trastuzumab

## Abstract

The objective of this multicenter, single‐arm trial (ChiCTR1900022293) was to explore the efficacy and safety of neoadjuvant therapy with epirubicin, cyclophosphamide, and pyrotinib followed by docetaxel, trastuzumab, and pyrotinib (ECPy‐THPy) in the treatment of patients with stage II–III HER2‐positive breast cancer. The present study enrolled patients with stage II–III HER2‐positive breast cancer. Epirubicin and cyclophosphamide were administrated for four 21‐day cycles, followed by four cycles of docetaxel and trastuzumab. Pyrotinib was taken orally once per day throughout the treatment period. The primary endpoint was total pathological complete response (tpCR, ypT0/is ypN0) rate in the modified intention‐to‐treat (mITT) population. In total, 175 patients were included. The tpCR rate was 68.6% (95% CI, 60.7–75.8%), while the objective response rate was 89.1%. In the post‐hoc subgroup analysis, no association between clinical characteristics and the tpCR rate was observed. The most common grade ≥3 adverse events were diarrhea (54.3%), followed by white blood cell count decreased (5.1%), and neutrophil count decreased (4.6%). In conclusion, the neoadjuvant regimen with ECPy‐THPy showed promising pathological response and clinical benefits with an acceptable safety profile in patients with stage II–III HER2‐positive breast cancer.

## Graphical abstract

In this multicenter, single‐arm trial, we investigated the efficacy and safety of the ECPy‐THPy regimen (epirubicin, cyclophosphamide, and pyrotinib followed by docetaxel, trastuzumab, and pyrotinib) as neoadjuvant therapy for patients with stage II–III HER2‐positive breast cancer. Our results revealed that tpCR was achieved in 107 (68.6%) out of 156 patients, which was in line with our previous pilot study and numerically higher than the tpCR rate in previous phase III KRISTINE trial and the phase III POENY trial.

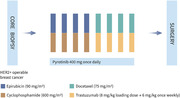

## 1 INTRODUCTION

Trastuzumab, a humanized monoclonal antibody targeting the human epidermal growth factor receptor 2 (HER2), when combined with chemotherapy in neoadjuvant or adjuvant setting, has remarkably improved response rates and survival outcomes of patients with HER2‐positive early or locally advanced breast cancer.^1^, ^2^, ^3^, ^4^, ^5^ However, a considerable subset of patients will develop trastuzumab resistance and suffer from disease relapse.^6^

Pyrotinib is an oral, irreversible pan‐HER tyrosine kinase inhibitor (TKI) that targets HER1, HER2 and HER4 by binding to the ATP‐binding region of the HER family.^7^ In China, pyrotinib combined with capecitabine has been approved for the treatment of HER2‐positive metastatic breast cancer patients who had received trastuzumab and taxane, based on results from the PHOEBE and the PHENIX trials.^8^, ^9^, ^10^, ^11^ Furthermore, the phase III PHILA trial showed that when compared with placebo plus trastuzumab and docetaxel, pyrotinib combined with trastuzumab and docetaxel markedly prolonged the median progression‐free survival in the first‐line setting, alongside a manageable safety profile.^12^

In terms of (neo) adjuvant treatment, for HER2‐positive breast cancer, the most commonly used chemotherapy regimens in clinical practice were taxanes plus platinum (TCb) and anthracycline plus cyclophosphamide followed by taxanes (AC‐T). AC‐T is a classic regimen, whether in single‐ or dual‐targeted anti‐HER2 (neo)adjuvant therapy for early‐stage breast cancer. It is supported by sufficient clinical evidence and is recommended in NCCN guidelines.^2^, ^13^, ^14^ However, cardiotoxicity seems decreased with platinum‐containing regimens (TCb).^15^ The phase III PHEDRA study demonstrated that the addition of pyrotinib to trastuzumab plus docetaxel significantly increased the pathological complete response (pCR) rate.^16^, ^17^ Nevertheless, the utilization of four cycles of single‐agent chemotherapy (docetaxel) in the PHEDRA study does not align with current clinical practice, prompting a need for identifying the optimal chemotherapy partner. While the NeoATP trial demonstrated the efficacy and safety of four cycles of pyrotinib and trastuzumab combined with weekly TCb neoadjuvant chemotherapy for patients with HER2‐positive locally advanced breast cancer, the study had a relatively small sample size. Besides, there is still a lack of exploration of the AC‐T chemotherapy regimen combined with trastuzumab and pyrotinib in the neoadjuvant setting. In our previous single‐center pilot trial, the combination of AC‐T chemotherapy regimen and trastuzumab plus pyrotinib achieved a relatively high pCR rate (14 out of 19, 73.7%).^14^ In the present study, we aim to verify these results in a multicenter design and with a larger sample size.

In the current multicenter, single‐arm study, our aim was to investigate the efficacy and safety of epirubicin, cyclophosphamide and pyrotinib followed by docetaxel, trastuzumab, and pyrotinib (ECPy‐THPy) for the neoadjuvant treatment of patients with HER2‐positive breast cancer.

## 2 RESULTS

### 2.1 Patient characteristics

**FIGURE 1:**
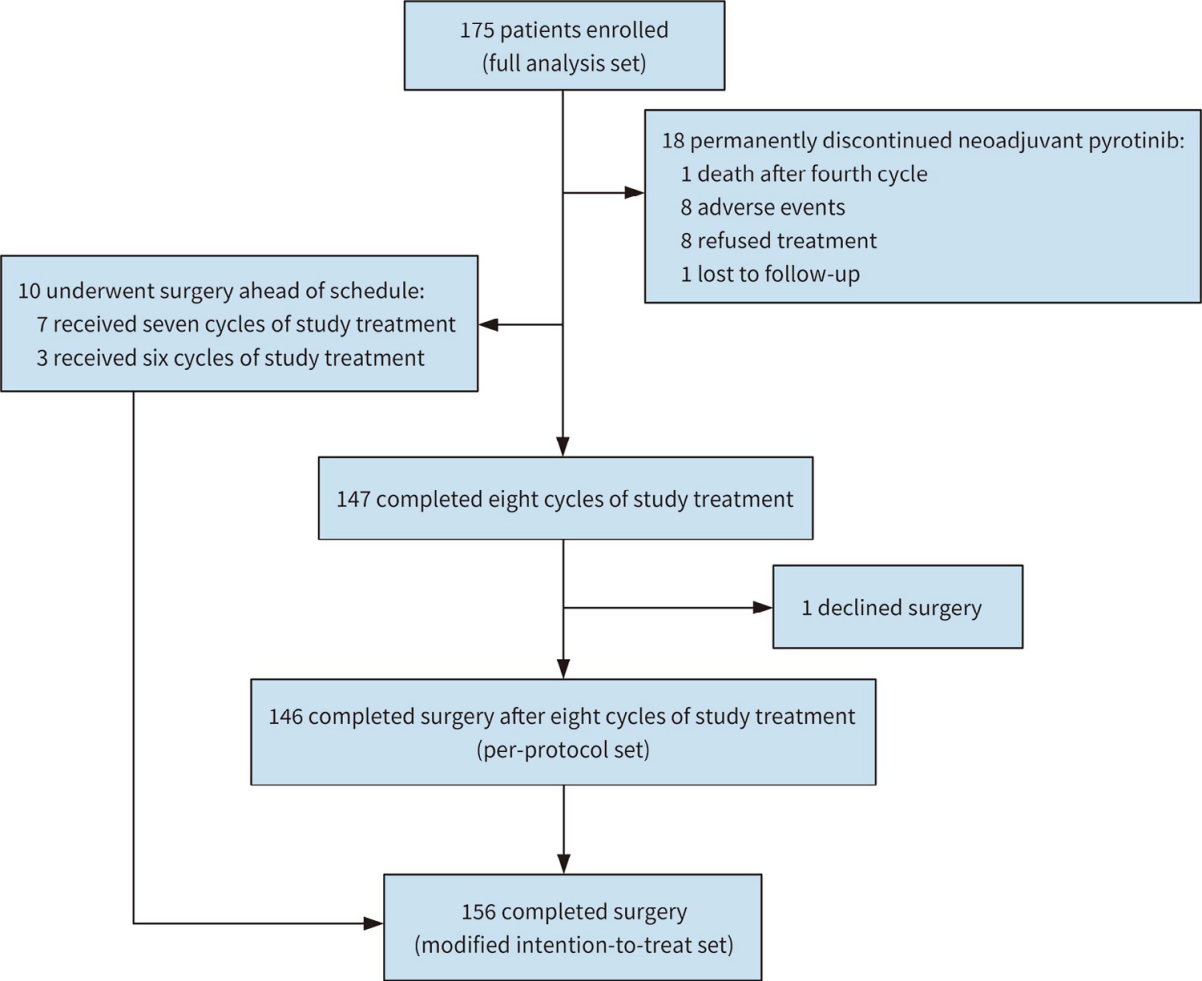
Trial profile of ChiCTR1900022293.

Between May 2020 and May 2022, a total of 175 patients were recruited from 16 centers in China (Table S1). Among them, 18 patients came off protocol and permanently discontinued neoadjuvant pyrotinib, and one patient declined surgery after neoadjuvant treatment. Finally, 156 patients underwent surgery with evaluable pathological data and were defined as modified intention‐to‐treat (mITT) population, among whom 10 patients failed to complete eight cycles of neoadjuvant treatment before surgery, resulting in 146 patients who received surgery after finishing study treatment (per‐protocol set). (Figure 1). Baseline characteristics of 175 participants were summarized in Table 1.

**TABLE 1:** Baseline characteristics of full analysis set (*n* = 175).

Characteristics	Full analysis set (*n* = 175)
Age (median[range]), years	51 [26, 68]
≤50, *n* (%)	86 (49.1)
>50, *n* (%)	89 (50.9)
Menopausal status, *n* (%)	
Premenopausal	83 (47.4)
Postmenopausal	92 (52.6)
Clinical T stage, *n* (%)	
cT0	1 (0.6)
cT1	11 (6.3)
cT2	130 (74.3)
cT3	29 (16.6)
cT4	4 (2.3)
Clinical N stage, *n* (%)	
cN0	49 (28.0)
cN1	94 (53.7)
cN2	11 (6.3)
cN3	21 (12.0)
Clinical TNM stage, *n* (%)	
IIA	52 (29.7)
IIB	75 (42.9)
IIIA	23 (13.1)
IIIB	4 (2.3)
IIIC	21 (12.0)
ER status, *n* (%)	
Positive	85 (48.6)
Negative	89 (50.9)
Unknown	1 (0.6)
PR status, *n* (%)	
Positive	64 (36.6)
Negative	111 (63.4)
HR status, *n* (%)	
Positive	96 (54.9)
Negative	79 (45.1)
HER2 status, *n* (%)	
3+	159 (90.9)
2+, FISH +	16 (9.1)
Ki67 level, *n* (%)	
≤30	100 (57.1)
>30	74 (42.3)
Unknown	1 (0.6)
TILs level (median [IQR]), %	27 (10‐44)
Low, *n* (%)	10 (5.7)
Intermediate, *n* (%)	18 (10.3)
High, *n* (%)	36 (20.6)
Unknown, *n* (%)	111 (63.4)

Abbreviations: ER, estrogen receptor; FISH, fluorescence in situ hybridization; HER2, human epidermal growth factor receptor 2; HR, hormone status; IQR, interquartile range; PR, progesterone receptor; TILs, tumor‐infiltrating lymphocytes.

The median age was 51 years (range: 26−68 years). The proportion of patients with lymph node metastasis was 72.0% (126 out of 175), and the distributions of clinical stage II and III were 72.6% (127 out of 175) and 27.4% (48 out of 175), respectively. The proportion of patients with a negative hormone receptor (HR) status was 45.1% (79 out of 175), and 42.3% (74 out of 175) of patients had high proliferative index (Ki67 > 30%). Tumor‐infiltrating lymphocytes (TILs) levels were only assessed in patients recruited from Southwest Hospital of Army Medical University. Therefore, only 64 (36.6%) tumor samples were evaluable. The median (interquartile range) level of TILs was 27% (10−44%).

### 2.2 Total pCR rate and subgroup analysis

**FIGURE 2:**
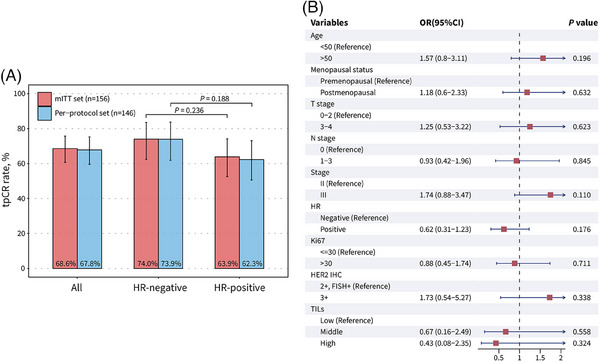
Total pathological complete response (tpCR) rate. (A) tpCR rate in modified intention‐to‐treat (mITT) set and per‐protocol set for the overall population and by hormone receptor (HR) status. Two‐sided *P* values are from chi‐square test. (B) Subgroup analysis of tpCR rates and odds ratios (ORs) of pCR in mITT set (*n* = 156). *p* Values and ORs are from univariate logistic regression. HR, hormone status; HER2, human epidermal growth factor receptor 2; IHC, immunohistochemical; FISH, fluorescence in situ hybridization. TILs, tumor‐infiltrating lymphocytes.

Collectively, the total pCR (tpCR) rate was 68.6% (107 out of 156; 95% confidence interval [CI], 60.7−75.8%) in the mITT set and 67.8% (99 out of 146; 95% CI, 59.6−75.3%) in the per‐protocol set (Figure 2A). Miller‐Payne grade 4 and 5 pathological responses were achieved in 24 (15.4%) and 107 (68.6%) patients, respectively (Table S2). A numerically higher tpCR rate in patients with HR‐negative disease was observed than those with HR‐positive disease, but this difference did not achieve statistical significance in both the mITT set (*p* = 0.236) and per‐protocol set (*p* = 0.188) (Figure 2A). In post‐hoc subgroup analysis, no association between clinical characteristics and tpCR were found, including TILs (Figure 2B).

### 2.3 Clinical response

**FIGURE 3:**
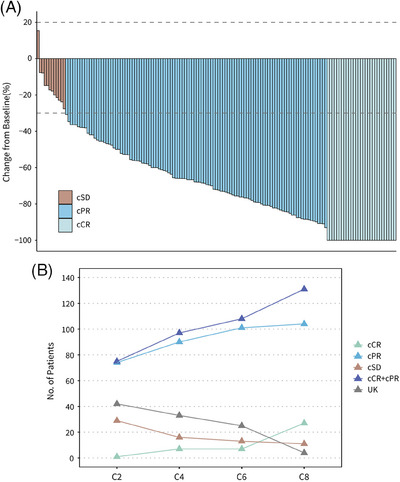
Clinical response by RECIST 1.1. (A) Waterfall plot of clinical response before surgery in mITT set (*n* = 156). (B) Best clinical response after two, four, six, and eight cycles of ECPy‐THPy in per‐protocol set (*n* = 146). cSD, clinical stable disease; cPR, clinical partial response; cCR, clinical complete response; UK, unknown; C2/4/6/8, cycle 2/4/6/8.

Regarding the clinical response to neoadjuvant therapy before surgery, 29 (18.6%) of 156 patients achieved clinical complete response (cCR), and 110 (70.5%) achieved clinical complete partial response (cPR), leading to an objective response rate (ORR) of 89.1% (95% CI, 83.1−93.5%) (Figure 3A and Table S3). No patient was evaluated as clinical progressive disease (cPD). The best clinical responses in the per‐protocol set were evaluated after two, four, six, and eight cycles of neoadjuvant chemotherapy, with 1 (0.7%), 7 (4.8%), 7 (4.8%), and 27 (18.5%) patients achieving cCR, respectively. The numbers of patients who achieved cCR or cPR after two, four, six, and eight cycles were 75 (51.4%,) 97 (66.4%), 108 (74.0%), and 131 (89.7%), respectively (Figure 3B and Table S4).

### 2.4 Safety

Of 175 patients with available safety data (safety set), 174 (99.4%) experienced treatment‐related adverse events (AEs) (Table 2). The most common AEs of any grade were diarrhea (94.3%), followed by anemia (79.4%), and vomiting (65.7%). The majority of patients (114, 65.1%) experienced grade ≥3 AEs, with diarrhea (54.3%), white blood cell count decreased (5.1%), and neutrophil count decreased (4.6%) being the most frequently reported. Serious AEs were reported in 3 (1.7%) patients, including acute kidney injury, pancreatitis, and hepatic failure. One patient died due to hepatic failure during neoadjuvant chemotherapy, most likely caused by the coexisting HBV infection. A total of 65 (37.1%) patients underwent pyrotinib dose adjustment due to AEs, among them, eight (4.6%) patients permanently discontinued neoadjuvant pyrotinib.

**TABLE 2:** Summary of treatment‐related adverse events (AEs).

AE	Any grade, *n* (%)	Grade 1, *n* (%)	Grade 2, *n* (%)	Grade 3, *n* (%)	Grade 4, *n* (%)	Grade 5, *n* (%)
Diarrhea	165 (94.3)	10 (5.7)	60 (34.3)	95 (54.3)	0	0
Anemia	139 (79.4)	80 (45.7)	52 (29.7)	7 (4.0)	0	0
Vomiting	115 (65.7)	49 (28.0)	61 (34.9)	5 (2.9)	0	0
Nausea	86 (49.1)	61 (34.9)	25 (14.3)	0	0	0
Hypoalbuminemia	76 (43.4)	74 (42.3)	2 (1.1)	0	0	0
Hand‐foot syndrome	67 (38.3)	49 (28.0)	18 (10.3)	0	0	0
Malaise	66 (37.7)	56 (32.0)	10 (5.7)	0	0	0
Anorexia	66 (37.7)	51 (29.1)	15 (8.6)	0	0	0
White blood cell decreased	63 (36.0)	32 (18.3)	22 (12.6)	8 (4.6)	1 (0.6)	0
Creatinine increased	62 (35.4)	59 (33.7)	3 (1.7)	0	0	0
Alanine aminotransferase increased	58 (33.1)	49 (28.0)	5 (2.9)	4 (2.3)	0	0
Alopecia	52 (29.7)	40 (22.9)	12 (6.9)	0	0	0
Mucositis oral	51 (29.1)	35 (20.0)	14 (8.0)	2 (1.1)	0	0
Weight loss	45 (25.7)	26 (14.9)	17 (9.7)	2 (1.1)	0	0
Aspartate aminotransferase increased	44 (25.1)	38 (21.7)	3 (1.7)	3 (1.7)	0	0
Neutrophil count decreased	41 (23.4)	20 (11.4)	13 (7.4)	6 (3.4)	2 (1.1)	0
GGT increased	40 (22.9)	32 (18.3)	5 (2.9)	3 (1.7)	0	0
Hypokalemia	36 (20.6)	30 (17.1)	2 (1.1)	4 (2.3)	0	0
Alkaline phosphatase increased	26 (14.9)	24 (13.7)	2 (1.1)	0	0	0
Platelet count decreased	7 (4.0)	6 (3.4)	0	1 (0.6)	0	0
Acute kidney injury	1 (0.6)	0	0	0	1 (0.6)	0
Pancreatitis	1 (0.6)	0	0	0	1 (0.6)	0
Hepatic failure	1 (0.6)	0	0	0	0	1 (0.6)

Any AE occurring in at least 10% of patients and all ≥3 grade AEs are reported. AEs was determined according to National Cancer Institute Common Terminology Criteria for Adverse Events version 5.0. Grade 1, mild; Grade 2, moderate; Grade 3, severe or medically significant but not immediately life‐threatening; Grade 4, life‐threatening consequences; Grade 5, death related to AE.

## 3 DISCUSSION

To our knowledge, this multicenter, open‐label, single‐arm study is by far the largest clinical trial to investigate the efficacy and safety of neoadjuvant pyrotinib in combination with trastuzumab and chemotherapy regimens for early‐stage breast cancer. The study met its primary endpoint, with 107 (68.6%) of 156 patients in the mITT set achieving tpCR, which was numerically higher than historical tpCR rates for dual‐targeted trastuzumab and pertuzumab neoadjuvant therapy (NeoSphere^18^: 45.8%, PEONY^19^: 39.3%, KRISTINE^20^: 56%, TRAIN‑2^21^: 68%, TRYPHAENA^22^: 51.9%, BERENICE^23^: 61.8%). However, the interpretation is limited by the nonrandomized nature of this study.

In our pilot trial of neoadjuvant ECPy‐THPy conducted in 2019, 14 (73.7%) of 19 patients achieved tpCR.^24^ However, the robustness of results was limited by the sample size. The current multicenter trial with an expanded sample size of 175 patients showed a numerically lower tpCR rate than the previous pilot trial, but still met our original expectation. In terms of combination of pyrotinib and trastuzumab plus chemotherapy regimens, the single‐arm phase‐II Panphila trial, which used a regimen of neoadjuvant trastuzumab and pyrotinib plus docetaxel and carboplatin, reported that 38 (55.1%) patients achieved pCR out of 69 patients with HER2‐positive early breast cancer.^25^ Moreover, in the single‐arm phase‐II NeoATP trial, the pre‐defined locoregional pCR (ypT0/is ypN0) was achieved in 39 (73.6%) of 53 patients with locally advanced HER2‐positive breast cancer who received four cycles of pyrotinib and trastuzumab with weekly paclitaxel–cisplatin as neoadjuvant treatment.^26^ Given the lack of head‐to‐head studies, no definite conclusion can be drawn regarding the optimal choice of chemotherapy combinations, but it provides a reference for clinical medication.

In terms of clinical response, in the present study, only 29 (18.6%) of 156 patients in mITT set achieved a cCR, while 107 (68.6%) patients achieved a pCR. This indicates that the residual foci of primary tumors still exist in most patients when assessed by imaging examination, even though there are no surviving tumor cells. The residual foci mostly originate from the processes such as inflammatory responses and incomplete macrophage phagocytosis in the primary lesion as a response to chemotherapy.^27^ Among patients who achieved cCR, 10.3% (3/29) still have pathological residual tumor cells. This phenomenon indicated the deficiency of conventional imaging technologies, which needs improvement or exploring other biomarkers.

In terms of HR status, HR‐positive patients had a numerically lower tpCR rate than HR‐negative patients, although no statistically significant difference was found (OR, 0.62 [95% CI, 0.31‐1.23], *p* = 0.176)). The result is in consistent with previous research on single or dual targeted anti‐HER2 neoadjuvant therapy,^18^, ^19^, ^26^, ^28^ indicating a limited response rate in HR‐positive/HER2‐positive (triple‐positive) breast cancers. For the treatment of such patients, the addition of CDK4/6 inhibitor to HER2‐targeted therapy and endocrine therapy may become a new treatment strategy. For example, the MUKDEN 01 study investigated the efficacy and safety of neoadjuvant therapy with pyrotinib and letrozole plus dalpiciclib in triple‐positive breast cancers, and this fully oral, chemotherapy‐free combination led to a promising pathological response and well‐tolerated safety.^29^

Of note, in the present study, there were no positive finding in the post‐hoc subgroup analysis of pCR, regardless of TILs level. In the NeoALTTO trial of neoadjuvant lapatinib and/or trastuzumab treatment,^30^ a positive association was found between levels of TILs greater than 5% and higher pCR rates (*p* = 0.01), as well as longer event‐free survival (*p* = 0.002). In the CLEOPATRA study involving advanced HER2‐positive breast cancers treated with docetaxel, trastuzumab, and pertuzumab or placebo,^31^ higher TILs levels was significantly associated with improved overall survival (*p* = 0.0014). However, in our study, TILs levels were not associated with the pCR rate in 64 samples with accessible TILs data (middle vs. low, odds ratio [OR], 0.67 [95% CI, 0.16–2.49], *p* = 0.558; high vs. low, OR, 0.43 [95% CI, 0.08–2.35], *p* = 0.324). This result is consistent with the Panphilia study with neoadjuvant regimen contained pyrotinib and trastuzumab.^25^ In that study, no significant difference in the pCR rate was found between TILs subgroups, regardless of using cut‐offs of 5% (*p* = 0.638) or 50% (*p* = 0.279). On the basis of above information, the host antitumor immunity, as represented by baseline TILs levels, may not be identified as a predictive factor of treatment response to pyrotinib and trastuzumab in neoadjuvant setting.^32^, ^33^, ^34^

In addition, we previously reported a significant relationship between PIK3CA mutations and lower pCR rate (adjusted *p*= 0.024) among 425 cancer‐related genes assessed by next‐generation sequencing of archived tumor blocks from 50 patients enrolled in this clinical trial.^35^ This finding is consistent with some previous reports. However, the result was puzzlingly opposite to the findings of NeoATP trial, which reported no statistically significant difference in terms of pCR between subgroups by PIK3CA status (*p* = 0.958).^26^ Therefore, we conducted a Higgins *I* ^2^ test to evaluate the heterogeneity, and the result showed high heterogeneity between two studies (*I* ^2^ = 83.1%, *p* = 0.015). The sources contributing to this heterogeneity were remained to be further studied, potentially due to differences in sequencing approaches, sample types, chemotherapy regimens and cycles, and so on.

Another major concern about this study is whether eight neoadjuvant cycles are necessary in the presence of pyrotinib. According to the 2020 NCCN guideline, the preferred neoadjuvant therapy for HER2‐positive breast cancer were four cycles of doxorubicin and cyclophosphamide followed by four cycles of paclitaxel plus trastuzumab (AC‐TH) with or without pertuzumab.^14^ In some previous studies, a 39% pCR rate in patients who underwent four cycles epirubicin and cyclophosphamide followed by four cycles paclitaxel and trastuzumab regimen, with 71.4% of patients achieving cCR or cPR before surgery.^36^ Additionally, a 60.7% pCR rate was observed in the cohort with four standard cycles of fluorouracil/epirubicin/cyclophosphamide followed by four cycles of docetaxel plus trastuzumab and pertuzumab.^23^ The clinical response in the per‐protocol set was evaluated every two cycles and the results indicated that the amounts of patients who achieved cCR were ascending (1, 7, 7, and 27, respectively), as well as the ORRs (51.4, 66.4, 74.0, and 89.7%, respectively). Hence, it can be recognized that even in the presence of continuous daily pyrotinib, about 14% (20/146) of patients achieved a cCR after six neoadjuvant cycles. Therefore, shortening the duration of neoadjuvant chemotherapy may lead to an inadequate response to therapy.

The safety profile of this study remained consistent with our pilot trial and other previous reports.^24^, ^25^, ^26^ The most common grade ≥3 AEs were diarrhea (54.3%), white blood cell count decreased (5.1%), and neutrophil count decreased (4.6%). When compared with regimens containing lapatinib neoadjuvant regimens, regimens containing pyrotinib yielded apparent numerically lower incidence of grade ≥3 hematological toxic effects (e.g., leucopenia, neutropenia), but numerically higher incidence of grade ≥3 diarrhea.^28^, ^37^, ^38^ The management of diarrhea has been well established in several previous studies. A population pharmacokinetic study showed that the concomitant use of montmorillonite could reduce the bioavailability of pyrotinib by 50.3%. Another retrospective cohort study demonstrated that loperamide‐based regimens achieved a favorable antidiarrheal effect (95.3–100%) compared with nonloperamide‐based regimens (e.g., montmorillonite).^39^, ^40^ Therefore, preventive treatment with loperamide proves beneficial in boosting patients' adherence and reducing the likelihood of dose discontinuation. No relevant cardiac events have emerged in this trial.

Our work had some limitations: the nonrandomized design, short period of follow‐up, and the unregulated assessment of pCR due to the absence of an independent review committee. What is more, the proportion of HER2 IHC 3+ exceeds 90%, which is relatively higher than the normal epidemiological incidence rate in China, indicating a selective bias during the enrollment process in our trial. However, no significant relationship between HER2 IHC status and tpCR rate (OR, 1.73 [95% CI, 0.54–5.27], *p* = 0.338) were found in subgroup analysis, indicating the effects of selective bias would be tiny. Furthermore, while the combination of docetaxel and carboplatin plus trastuzumab with or without pertuzumab have become the preferred regimen for neoadjuvant therapy in HER2‐positive breast cancer according to the 2022 NCCN guideline, at the time of design of this study in 2019, AC‐TH with or without pertuzumab were preferentially recommendations in NCCN guideline.^14^ However, the combination of AC‐TH with or without pertuzumab still remains an alternative recommended regimen in the newest version of the NCCN guideline that released in June 2022.

In conclusion, pyrotinib and trastuzumab plus chemotherapy showed favorable therapeutic efficacy and an acceptable safety profile in patients with stage II–III HER2‐positive breast cancer in neoadjuvant setting.

## 4 METHODS

### 4.1 Study design and participants

This multicenter, open‐label, single‐arm clinical trial was carried out at 16 institutions in China from May 2020 to May 2022. Patients were eligible if they were: female aged 18−70 years; had histologically confirmed HER2‐positive invasive breast cancer; was clinical stage II–III according to the 8th Edition American Joint Committee on Cancer Breast Cancer Staging Manual; had an Eastern Cooperative Oncology Group (ECOG) Performance Status of 0 or 1; had at least one radiographically measurable lesion per the Response Evaluation Criteria in Solid Tumors, version 1.1 (RECIST 1.1); had adequate organ function at baseline, laboratory indicators include absolute neutrophil count, blood platelet, hemoglobin, total bilirubin, alanine aminotransferase, aspartate aminotransferase, left ventricular ejection fractions (LVEF), QT interval corrected by Fridericias formulae.

The current study was administrated in accordance with the Declaration of Helsinki and the Guidelines for Good Clinical Practice (identifier: ChiCTR1900022293). The study protocol obtained approval at each participating institution, and written informed consent was obtained from all participants before study entry.

### 4.2 Procedures

**FIGURE 4:**
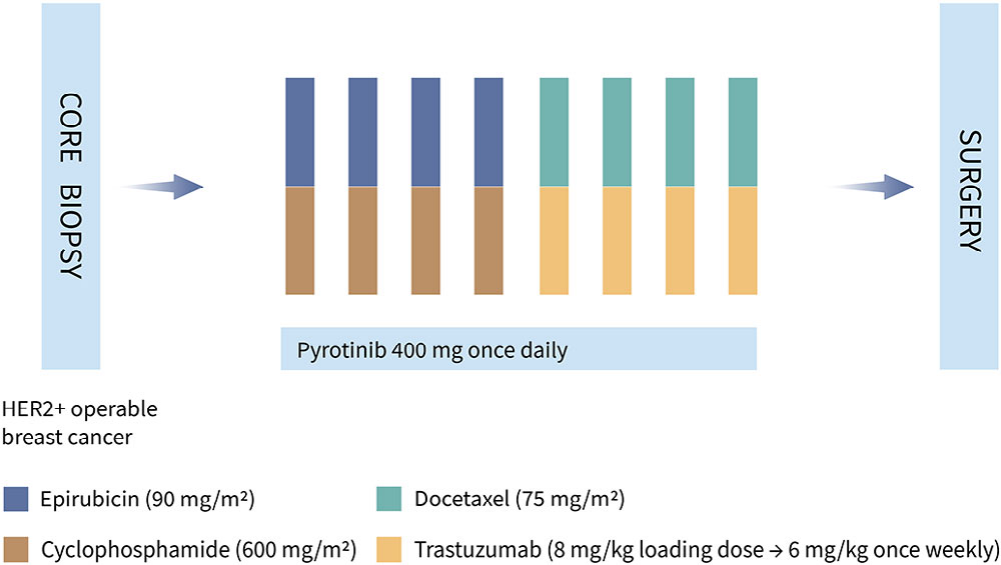
Schema of ChiCTR1900022293. HER2, human epidermal growth factor receptor 2.

As shown in Figure 4, four cycles of epirubicin (90 mg/m^2^, intravenously, day 1) and cyclophosphamide (600 mg/m^2^, intravenously, day 1), followed by four cycles of docetaxel (75 mg/m^2^, intravenously, day 1) and trastuzumab (8 mg/kg loading dose, followed by 6 mg/kg, intravenously, day 1) were performed in a 21‐day cycle. Pyrotinib (400 mg, orally, once per day) was given throughout the neoadjuvant therapy period until intolerable toxicity, disease progression, or withdrawal of consent at the patient's request. Dosage reduction of pyrotinib was allowed for toxicity, from 400 to 320 mg to a minimum dose of 240 mg daily. Permanent discontinuation of pyrotinib will be conducted if patients experienced grade ≥2 left ventricular systolic dysfunction or grade ≥4 diarrhea.

Surgery was conducted within 16–20 days after the last neoadjuvant therapy. If tpCR is achieved after postoperative pathological evaluation, trastuzumab combined with pyrotinib as adjuvant therapy will be continued for 1 year. Otherwise, the adjuvant strategy will be changed according to the clinician's recommendation (trastuzumab plus pertuzumab or T‐DM1).

The clinical response was determined by comparing the largest tumor diameter at baseline and every two cycles during therapy, as assessed by ultrasonography and magnetic resonance imaging (MRI), according to RECIST 1.1 criteria. The resected breast tissues at surgery were evaluated for pathological response with Miller‐Payne grading system. Safety assessment was conducted at each cycle, including the vital signs, ECOG status, 12‐lead electrocardiograms, hematologic and biochemical examination. LVEF was evaluated before study entry and repeated after 4 cycles and at the completion of treatment. AEs profiles including the type, incidence, and severity grade were compiled according to the National Cancer Institute Common Terminology Criteria for Adverse Events, version 5.0.

### 4.3 Endpoints

The primary endpoint was the investigator‐assessed tpCR (ypT0/is, ypN0) rate, defined as the proportion of patients with no residual cancer cells in both the excised breast specimen and all ipsilateral lymph node samples. The secondary endpoints included ORR, defined as the proportion of patients achieving cCR or cPR as per RECIST 1.1. Disease recurrence and survival outcomes will be analyzed after a median follow‐up time of approximately 60 months.

### 4.4 TILs evaluation

TILs are immunological biomarkers associated with anti‐tumor immunity. According to the recommendations of the international TILs working group in 2014, TILs were categorized as low (0−9%), intermediate (10−49%), and high (≥50%) by calculating the percentage of area occupied by mononuclear cells (including lymphocytes and plasma cells) in the stromal tissue area. Archived biopsy samples were stained with hematoxylin and eosin.^41^

### 4.5 Statistical analysis

According to historical reports, the pCR rate of dual‐targeted trastuzumab and pertuzumab neoadjuvant therapy is hypothesized to be 58%, and an expected pCR rate of 68% was established based on our previous pilot study.^20^, ^23^, ^24^ With an alpha level set at 0.05, a sample size of 157 participants was required for a power of 0.8 to detect an increase in the pCR rate using a two‐sided binomial test for superiority. Considering a dropout rate of 10%, a total of 174 participants were required. Baseline descriptive analysis of patient characteristics was conducted in the full analysis set, which included all patients treated with at least one dose of study drugs. Endpoints were assessed in the mITT population (comprising all patients who received at least one dose of study drugs and underwent surgery with evaluable pathological results), as well as the per‐protocol populations (patients who were compliant with the study protocol). These analyses were performed both overall and within HR subgroups. Safety was analyzed in the safety set, which included all patients who received at least one dose of study drugs and had available safety records. All primary and secondary endpoints were estimated with two‐sided CIs using the Clopper‐Pearson method for both the whole population and the subgroups. Post‐hoc subgroup analyses for pCR rate were conducted on the basis of baseline characteristics by using univariate logistic regression with a forest plot of ORs. Analyses and visualization were done with R, version 4.2.1.

## AUTHOR CONTRIBUTIONS

J. J., Y. Z., and X. Q. conceived the study. X. Q., P. T., and Q. S. contributed to the study design. M. W., H. C., P. L., D. C., J. H., Q. L., Y. Z., K. Y., Y. Z., C. C., Z. Z., L. Q., J. Z., M. M., and Y. S. contributed as subinvestigators. P. T., L. F., L. C., S. W., Y. L., Y. H., M. W., L. R., G. Z., X. T., L. Y., J. D., and X. W. coordinated samples and clinical data. Q. S. performed the statistical analysis. Q. S. and X. Q. drafted the manuscript. All authors read and approved the final manuscript.

## CONFLICT OF INTEREST STATEMENT

The authors declare no conflicts of interests.

## ETHICS STATEMENT

Study protocols were approved by the Ethical Review Community of Southwest Hospital (Approval Number: KY201922, Amendment Approval Number: KY2020084 and KY20201072). The ethics committee at each participating institution approved the study protocol. The study has been registered in The Chinese Clinical Trial Registry Registered on April 3, 2019 (Clinical Registration Number: ChiCTR1900022293).

## Supporting information

Supporting Information

## Data Availability

The raw clinical data are protected due to patient privacy laws. The deidentified datasets generated and/or analyzed during the current study are available from the corresponding author upon reasonable request, on the premise of the approval of the Institutional Ethical Committees. All the code programmed during this study are available from the corresponding author when requesting sharing.
