# Supplementary material for: A multicenter single‐arm trial of neoadjuvant pyrotinib and trastuzumab plus chemotherapy for HER2‐positive breast cancer
Source: MedComm (2020). 2023 Dec 7;4(6):e435. doi: 10.1002/mco2.435 (PMC10701463; doi:10.1002/mco2.435)
Supplement: Supplementary file 1 — Supporting Information [file MCO2-4-e435-s001.docx]

**Title page**

**A multicenter single-arm trial of neoadjuvant pyrotinib and trastuzumab plus chemotherapy for HER2-positive breast cancer**

**Running title:** Neoadjuvant pyrotinib for HER2+ BC

Qiyun Shi, PhD^1,2†^, Xiaowei Qi, MD^1†^, Peng Tang, MD^1^, Linjun Fan, MD^1^, Li Chen, MD^1^, Shushu Wang, MM^1^, Yan Liang, MD^1^, Ying Hu, MD^1^, Minghao Wang, MD^1^, Lin Ren, MM^1^, Guozhi Zhang, MM^1^, Xuanni Tan, MM^1^, Long Yuan, MD^1^, Junze Du, MM^1^, Xiujuan Wu, MD^1^, Mengyuan Wang, MD^3^, Hongying Che, MD^4^, Pengwei Lv, MD^5^, Dejie Chen, MD^6^, Jinhui Hu, MD^7^, Qiuyun Li, MD^8^, Yanwu Zhang, MD^9^, Kunxian Yang, MD^10^, Yuan Zhong, MD^11^, Chuang Chen, MD^12^, Zemin Zhou, MD^13^, Liyuan Qian, MD^14^, Jingwei Zhang, MD^15^, Mingde Ma, MD^16^, Yi Sun, MD^17^, Yi Zhang, MD^1*^, Jun Jiang, PhD, MD^1*^

^1^Department of Breast and Thyroid Surgery, Southwest Hospital, Army Medical University

^2^The Eighth Medical Center of Chinese PLA General Hospital

^3^Department of Breast surgery, Chongqing University Three Gorges Hospital

^4^Department of Thyroid and Breast Surgery, Zigong First People's Hospital

^5^Department of Breast surgery, The First Affiliated Hospital of Zhengzhou University

^6^Department of General Surgery, Xiangyang Central Hospital

^7^Department of Breast Surgery, The First Hospital of Hunan University of Chinese Medicine

^8^Department of Breast Surgery, Guangxi Medical University Cancer Hospital

^9^Department of Breast Surgery, The Third Affiliated Hospital of Zhengzhou University

^10^Department of Breast and Thyroid Surgery, The First People's Hospital of Yunnan Province

^11^Department of Breast and Thyroid Surgery, The Central Hospital of Wuhan

^12^Department of Breast and Thyroid Surgery, Hubei General Hospital

^13^Department of Breast and Thyroid Surgery, Huaihua First People's Hospital

^14^Department of Breast and Thyroid surgery, The Third Xiangya Hospital of Central South University

^15^Department of Breast and Thyroid Surgery, Zhongnan Hospital of Wuhan University

^16^Department of Thyroid and Breast surgery, Huaihe Hospital of Henan University

^17^Department of Breast and Thyroid Surgery, Xuchang Central Hospital

†Qiyun Shi and Xiaowei Qi have contributed equally to this work

***Correspondence:**

Yi Zhang: [ZY53810@163.com](mailto:ZY53810@163.com);

Jun Jiang: jcbd@medmail.com.cn.

Department of Breast and Thyroid Surgery, Southwest Hospital, Army Medical University

Mailing address: No. 30 Gaoyantan Street, Shapingba District, Chongqing, 400038, People’s Republic of China

Tel: +86-023-65318301

**Table S1**. Study sites, principal investigators and number of recruited patients.

| Study site | Principal investigator | No. of recruited patients |
| --- | --- | --- |
| Department of Breast and Thyroid Surgery, Southwest Hospital, Army Medical University | Jun Jiang, Yi Zhang | 85 |
| Department of Breast surgery, Chongqing University Three Gorges Hospital | Mengyuan Wang | 25 |
| Department of Thyroid and Breast Surgery, Zigong First People's Hospital | Hongying Che | 23 |
| Department of Breast surgery, The First Affiliated Hospital of Zhengzhou University Zhengzhou | Pengwei Lv | 9 |
| Department of General Surgery, Xiangyang Central Hospital | Dejie Chen | 8 |
| Department of Breast Surgery, Guangxi Medical University Cancer Hospital | Qiuyun Li | 5 |
| Department of Breast Surgery, The First Hospital of Hunan University of Chinese Medicine | Jinhui Hu | 4 |
| Department of Breast Surgery, The Third Affiliated Hospital of Zhengzhou University | Yanwu Zhang | 4 |
| Department of Breast and Thyroid Surgery, The First People's Hospital of Yunnan Province | Kunxian Yang | 2 |
| Department of Breast and Thyroid Surgery, The Central Hospital of Wuhan | Yuan Zhong | 2 |
| Department of Breast and Thyroid Surgery, Hubei General Hospital | Chuang Chen | 2 |
| Department of Breast and Thyroid Surgery, Huaihua First People's Hospital | Zemin Zhou | 2 |
| Department of Breast and Thyroid surgery, The Third Xiangya Hospital of Central South University | Liyuan Qian | 1 |
| Department of Breast and Thyroid Surgery, Zhongnan Hospital of Wuhan University | Jingwei Zhang | 1 |
| Department of Thyroid and Breast surgery, Huaihe Hospital of Henan University | Mingde Ma | 1 |
| Department of Breast and Thyroid Surgery, Xuchang Central Hospital | Yi Sun | 1 |

**Table S2**: The histologic response assessed by the Miller-Payne grading system.

| Variable | Efficacy-evaluable set (n = 156) | Per-protocol set (n = 146) |
| --- | --- | --- |
| tpCR | 107 (68.6) | 99 (67.8) |
| Miller-Payne grade, n (%) |  |  |
| Grade 5 (bpCR) | 107 (68.6) | 99 (67.8) |
| Grade 4 | 24 (15.4) | 23 (15.8) |
| Grade 3 | 17 (10.9) | 17 (11.6) |
| Grade 2 | 7 (4.5) | 6 (4.1) |
| Grade 1 | 1 (0.6) | 1 (0.7) |

tpCR, total pathological complete response; bpCR, breast pathological complete response.

**Table S3**: Clinical response to neoadjuvant therapy before surgery in the modiﬁed intention-to-treat (mITT) set (n = 156).

| Variable | mITT set (n=156) |
| --- | --- |
| Clinical response, n (%) |  |
| cCR | 29 (18.6) |
| cPR | 110 (70.5) |
| cSD | 12 (7.7) |
| cPD | 0 |
| UK | 5 (3.2) |
| ORR, n (%) | 139 (89.1) |

cCR, clinical complete response; cPR, clinical partial response; cSD, clinical stable disease; cPD, clinical progressive disease; UK, unknown; ORR, objective response rate.

**Table S4**: Best clinical response after two, four, six, and eight cycles of study treatment in per-protocol set (n = 146).

| Variable | Cycle 2 | Cycle 4 | Cycle 6 | Cycle 8 |
| --- | --- | --- | --- | --- |
| Clinical response, n (%) |  |  |  |  |
| cCR | 1 (0.7) | 7 (4.8) | 7 (4.8) | 27 (18.5) |
| cPR | 74 (50.7) | 90 (61.6) | 101 (69.2) | 104 (71.2) |
| cSD | 29 (19.9) | 16 (11.0) | 13 (8.9) | 11 (7.5) |
| cPD | 0 | 0 | 0 | 0 |
| UK | 42 (28.8) | 33 (22.6) | 25 (17.1) | 4 (2.7) |
| ORR, n (%) | 75 (51.4) | 97 (66.4) | 108 (74.0) | 131 (89.7) |

cCR, clinical complete response; cPR, clinical partial response; cSD, clinical stable disease; cPD, clinical progressive disease; UK, unknown; ORR, objective response rate.
